# Comparative analysis of phytocompounds and repurposed drugs against dengue virus serotypes employing an in silico study

**DOI:** 10.1038/s41598-025-06974-y

**Published:** 2025-07-30

**Authors:** Pranjal Vats, Rajan Rolta, Deeksha Salaria, Ishika Sharma, Anita Verma, Olatomide A. Fadare, Mansi Verma

**Affiliations:** 1 School of Biological Sciences, The University of Manchester, Oxford Road, Manchester, M13 9PL UK; 2 Department of Pharmacology, Post Graduate Institute of Medical Education and Research, Chandigarh, 160012 India; 3 Sri Venkateswara College, University of Delhi, New Delhi, 110021 India; 4 Organic Chemistry Research Lab, Department of Chemistry, ObafemiAwolowo University, Ile-Ife, Osun 220282 Nigeria; 5 Department of Zoology, Hansraj College, University of Delhi, Delhi, 110007 India

**Keywords:** DENV, Phytocompounds, Repurposed drugs, Serotypes, MD simulation, Chemical biology, Computational biology and bioinformatics, Microbiology, Diseases, Health care

## Abstract

Dengue virus (DENV) has emerged as a formidable global health challenge, with a surging incidence rate across the world. Despite numerous research initiatives aimed at developing effective antiviral therapy, no clinically proven drug or vaccine has been identified to combat all four genetically diverse serotypes of DENV. Therefore, comparative analysis of repurposed drugs and phytocompounds against all DENV serotypes is critical in the search for an effective long-term solution to this menacing disease. 93 phytocompounds and 15 drugs were shortlisted from the literature and screened using DataWarrior 5.5.0, from which 10 phytocompounds and 10 drugs were selected for further analysis. Molecular docking was performed by using AutoDockVina tool. Toxicity and druglikeness activity of standard drugs and phytoconstituents was done by using online servers. The current study showed that among all the selected phytoconstituents, lupiwighteone showed the best binding energy, favorable pharmacokinetics and no toxicity with all the selected serotypes of DENV. The MD simulation result supported the stability of lupiwighteone in complexes with NS3, NS5 and E-protein. This study identifies lupiwighteone as a promising antiviral candidate with favorable drug-like properties against DENV-2, DENV-3, and DENV-4 serotypes. Furthermore, in vitro and in vivo study is required for the validation of antiviral activity of lupiwighteone against dengue virus.

**Supplementary Information:**

The online version contains supplementary material available at 10.1038/s41598-025-06974-y.

## Introduction

Dengue Virus (DENV), belonging to the *Flaviviridae* family, is one of the rapidly growing arthropod-borne viral diseases which has become a primary concern of public health globally^1–3^. WHO reported an 8-fold increase in dengue cases between 2000 and 2019^2^. This rise, which is most noticeable in many tropical and subtropical countries, is the result of inadequate mosquito control, climate change, resistant mosquito emergence, unplanned urbanization, and a significant increase in population. DENV is transmitted by mosquitoes of the *Aedes* genus, essentially *Aedes aegypti*^3,4^. Clinical features of dengue virus infection can range from being asymptomatic to mild febrile illness and to having severe dengue haemorrhagic fever (DHF)^5^. But the treatment has been difficult due to the lack of effective antiviral therapy, as well as the ordeal of accurately diagnosing the disease due to its diverse presentation. Moreover, the use of these therapies in the presence of non-neutralizing antibodies may actually increase the risk of antibody-dependent enhancement (ADE), worsening the symptoms of dengue fever^1,6^. As a result, several initiatives in the domains of computational biology and drug docking have been undertaken to use medicinal phytocompounds and repurposed pharmaceuticals against DENV serotypes.

Understanding the genomics and proteomics of dengue at the serotype level is essential to effectively combat this virus. In 2019, our study delved into exploring conserved epitopes across all four serotypes and revealed 100% conservation in the Envelope (E) protein, NS3, NS4A, NS4B, and NS5 proteins^1^. Subsequently, in 2022, we investigated the conservancy in genomic regions of evolutionarily significant sequences, focusing on CpG islands spanning all serotypes. Our findings demonstrated that three DENV proteins (NS5, NS3, and E) exhibit highly conserved motifs across all four serotypes^7^. Thus, based on the previous studies, present investigation focused on NS5, NS3, and E proteins due to their consistency across serotypes. NS5 is an integral and highly conserved protein, with RNA-dependent RNA polymerase (RdRp) and methyltransferase activities, making NS5 a promising target for several anti-DENV medications^8^. Similarly, NS3, a multifunctional protein; and E surface Envelope protein are also additional potent drug targets^9–11^. Due to high mutation rates in RNA viruses and high genetic diversity, targeting conserved proteins of DENV may result in consistent binding of phytochemicals and drugs with all serotypes^12^. Thus, these three proteins become the most attractive targets for vaccines and antiviral drugs. In this study, a comparative analysis of all serotypes of DENV is done against some of the repurposed drugs and phytochemicals, thus aiding in the selection of new therapeutics effective for all serotypes.

Various studies reported that the phytocompounds extracted from medicinal plants like *Thymus serpyllum*, *Cymbopogan citratus*, *Foeniculum vulgare*, *Bacopa monnieri*, *Rheum emodi* have demonstrated a broad spectrum of pharmacological properties against various diseases^13–22^. On the other hand, drug repurposing is a viable approach for developing new therapeutic applications while minimising adverse side effects and cytotoxicity. Existing pharmacological compounds are carefully examined to uncover new therapeutic uses. The use of phytocompounds also offers a compelling array of benefits, emphasised by their perfect safety profile, which includes low toxicity potential and a remarkably low level of detrimental effects. As a result, phytocompounds indicate they are potential candidates for the development of therapeutic interventions that are both efficient and well-tolerated. Phytocompounds like naringin, maslinic acid, steroic acid, and rhodiolin have been successfully docked with DENV’s NS2B/NS3 protease and NS5 methyltransferase in previous studies^23,24^. However, a comparative analysis of phytocompounds across all serotypes requires further attention. Repurposed drugs like chloroquine, prednisone and lovastatin, to name a few, have shown limited efficacy in reducing viremia during clinical evaluation^25^, necessitating the identification of more robust therapeutic alternatives. Several inhibitors targeting key DENV proteins, such as NS3 protease, NS5 and the Envelope (E) protein, have been reported in literature including natural phytocompounds, synthetic small molecules, and repurposed drugs. Inhibitors like (1S,5R)-3-methyl-2-thioxo-1,2,3,4,5,6-hexahydro-8H-1,5-methanopyrido[1,2-a][1,5]diazocin-8-one (E-protein inhibitor); (2S,4S)-4-(2-Hydroxyphenyl)-2-(methylthio)-1,2,3,4-tetrahydropyrrolo[1,2-a]quinolin-3-one (NS3 inhibitor); 2-Oxo-1,3-benzoxathiol-5-yl acetate (NS5 inhibitor) of DENV have shown promising in silico or in vitro activity^26–28^. However, many of these studies are limited to individual serotypes or lack validation through dynamic simulations, highlighting the need for a more comprehensive and comparative analysis across all four DENV serotypes. In this study, a comparative analysis of all serotypes of DENV is done against some of the repurposed drugs and phytocompounds, thus aiding in the selection of new therapeutics effective for all serotypes.

## Methods

### Ligand preparation

**Fig. 1:**
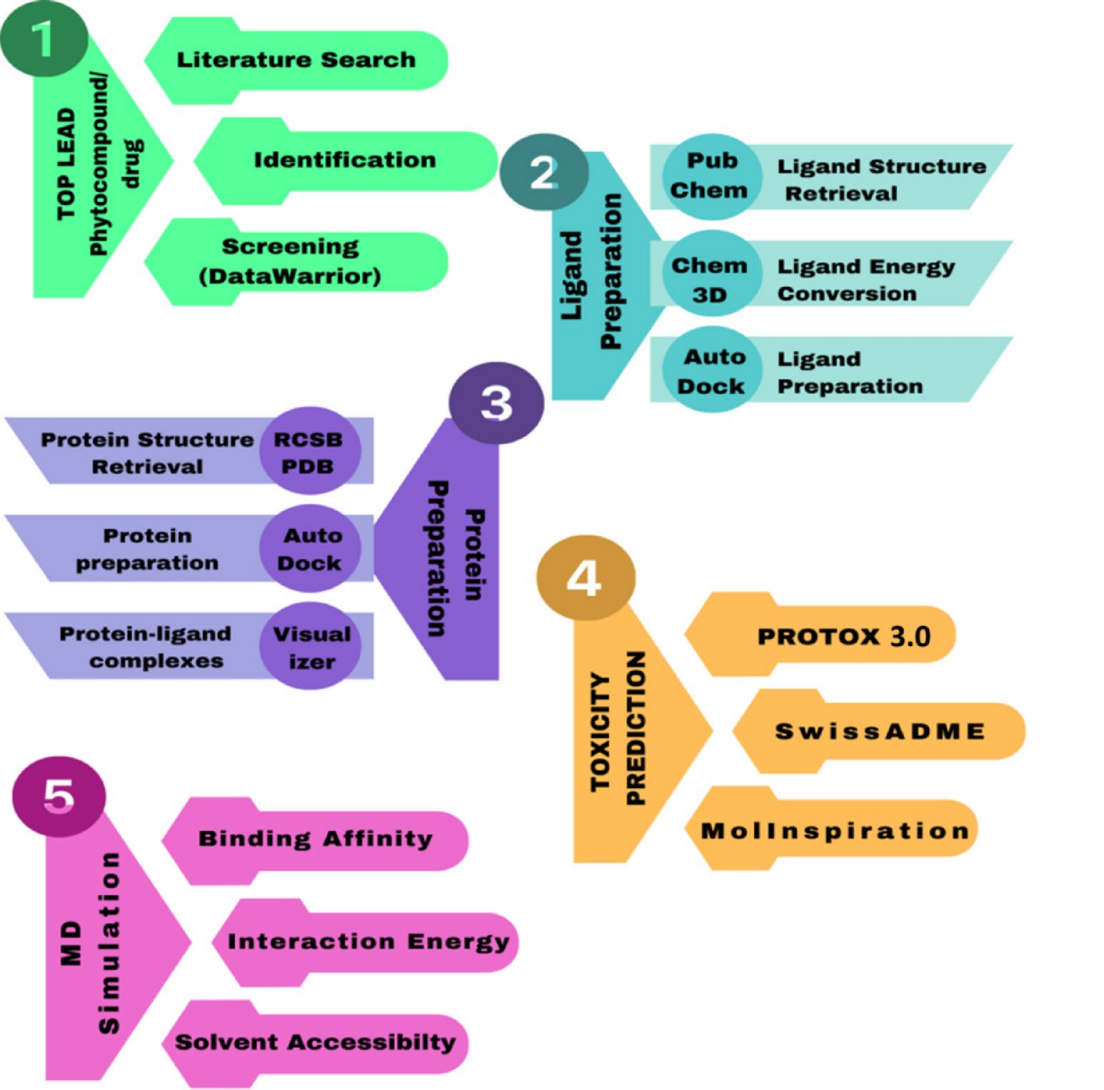
Schematic flowchart illustrating the comprehensive methodology.

The investigation began with a comprehensive review of the literature which resulted in identifying 93 phytoconstituents and 15 standard drugs with purported antiviral properties. Employing the DataWarrior 5.5.0 tool (https://openmolecules.org/datawarrior), a meticulous screening process was initiated to discern the most promising compounds. Following an exhaustive analysis of the generated data, the top 10 phytocompounds and 10 drugs were curated for subsequent evaluation (Fig. 1).

To facilitate molecular docking simulations, the three-dimensional structures of the selected phytoconstituents and standard drugs were procured from PubChem (https://pubchem.ncbi.nlm.nih.gov/) and DrugBank (https://go.drugbank.com/), in .sdf format. These structures were then subjected to rigorous energy minimization using Chem3D 15.0 to refine their conformations and enhance stability. Following this optimization step, the ligands were converted to PDB format to ensure compatibility with subsequent docking procedures.

The prepared ligands underwent further refinement using AutoDock Tools 1.5.6 to ensure proper configuration and compatibility with the docking software. Subsequently, the ligand structures were saved in pdbqt format, a prerequisite for conducting accurate and reliable docking simulations. This meticulous preparation of the ligands laid a solid foundation for robust and insightful molecular docking studies.

### Preparation of target proteins

Based on the availability of 3D crystal structures, the protein structures for NS3 of DENV-4 (PDB ID: 2VBC)^29^ and DENV-2 (PDB ID: 2FOM)^30^; NS5 of DENV-3 (PDB ID: 4V0Q)^31^ and DENV-2 (PDB ID: 5ZQK)^32^; and E-protein of DENV-2 (PDB ID: 1OKE)^33^ were retrieved from the RCSB-PDB database (https://www.rcsb.org/). These structures were saved in PDB format for further analysis.

To prepare the protein structures for docking simulations, AutoDock Tools 1.5.6 was employed. This involved the removal of water molecules and the addition of missing hydrogen atoms and charges to ensure the accuracy of the protein models. The prepared protein structures were then saved in pdbqt format, rendering them compatible with subsequent docking procedures.

The grid box for each protein was set over the entire protein (blind docking) using the default center coordinates and extending the box size until the entire protein is covered in most cases: for NS5 of DENV-2 (x = 33.572, y=-11.688, z = 25.66), NS5 of DENV-3 (x = 24.892, y = 162.151, z = 24.605), NS3 of DENV-2 (x = 0.455, y=-16.942, z = 13.968), NS3 of DENV-4 (x=-6.068, y=-2.850, z = 20.867), and for E of DENV-2 (x=-13.129, y = 69.049, z = 24.451).

### Molecular Docking

Molecular docking analysis was conducted using the AutoDockVina tool^34^ to investigate the interactions between the selected ligands and the active sites of the target proteins with the exhaustiveness set to “8” in the configuration file. This approach aimed to elucidate the nature and causes of the interactions between the sketched compounds and the protein targets.

The docking study encompassed all prepared phytocompounds and drugs against the three target proteins of DENV. Subsequently, the most favorable conformations, characterized by the highest binding energies, were selected. The 10 compounds with the highest affinity for target protein for each docking run were selected. Protein-ligand complexes were then analyzed using Discovery Studio Visualizer to examine the array of interactions between the proteins and ligands.

Furthermore, the compounds demonstrating the most promising interactions with respective protein were chosen for further analysis for their Absorption, Distribution, Metabolism, Excretion, and Toxicity (ADMET) properties. This comprehensive approach provided insights into the potential therapeutic efficacy and safety profiles of the selected compounds.

### In silico toxicity prediction of phytocompounds and drugs

ADMET screening was conducted to evaluate the absorption, toxicity, and drug-likeness properties of the ligands. The analysis utilized several tools, including SwissADME (http://www.swissadme.ch), MolInspirationCheminformatics (https://www.molinspiration.com), and ProTox 3.0 (https://tox-new.charite.de/protox3/).

SwissADME was employed to identify predictive models for the physicochemical properties, drug-likeness, medicinal chemistry, and pharmacokinetics of the compounds. This facilitated the selection of compounds with optimal characteristics for inhibiting the action of DENV proteins. To further assess the safety profile of the compounds, their structures were uploaded to the ProTox 3.0 to predict toxicity endpoints such as cytotoxicity, hepatotoxicity, carcinogenicity, and mutagenicity. LD50 values were also considered for quantitative analysis.

Additionally, the MolInspiration server was utilized to predict bioactivity scores and assess molecular descriptors and druglikeliness properties. This analysis was based on Lipinski’s Rule of Five^21,35^, which stipulates that for a compound to be orally active, it should not violate more than one of the five criteria.

### Molecular dynamics (MD) simulation

The MD simulation of the protein-ligand complex was done using GROMACS 2021.5^36^ software which was installed in ubuntu 18.04 LTS on a GPU enabled PC having the nvidiaGeforce RTX 2070 maxQ graphics card. Simulations were conducted for complexes involving lupiwighteone with all three proteins,(1S,5R)-3-methyl-2-thioxo-1,2,3,4,5,6-hexahydro-8H-1,5-methanopyrido[1,2-a][1,5]diazocin-8-one (E-protein inhibitor); (2S,4S)-4-(2-Hydroxyphenyl)-2-(methylthio)-1,2,3,4-tetrahydropyrrolo[1,2-a]quinolin-3-one (NS3 inhibitor); 2-Oxo-1,3-benzoxathiol-5-yl acetate (NS5 inhibitor) to evaluate their structural stability and dynamic interactions. Docked structures of the protein-ligand complexes were used as starting points in the simulation study. Proteins were processed and the topology files were prepared by using pdb2gmx and charmm36 force field, while the ligands topology files were prepared by using the online server, SwissParam. The solvent (using the TIP3P water model) addition was done in a cubic box by using a box distance 1.0 nm from closest atom in the protein. The addition of Cl^−^ ions was used to neutralize the system. Energy minimization was done by using steepest descent algorithm taking 50,000 steps, 5 kJ/mol maximum force and Verlet cut-off scheme taking Particle-Mesh Ewald (PME) columbic interactions. Position restraints were applied in the equilibration step. After that, NVT equilibration was done in 300 K and 100 ps in 50,000 steps and NPT equilibration taking Parrinello-Rahman (pressure coupling), 1 bar reference pressure and 100 ps in 50,000 steps. LINCS algorithm was applied to constraint all the bond lengths. For long-range electrostatics, Particle-mesh Ewald (PME) algorithm was used. After successful completion of molecular dynamic simulation for 100 ns, Root mean square deviation (RMSD) of backbone residues, number of Hydrogen bonds, Root mean square fluctuations (RMSF), Radius of gyration (RG) and solvent accessible surface area (SASA)were calculated from the trajectory files from each complex.

The free energy of binding calculations was done using the standalone program, gmx_MMPBSA^37^ based on the MM-PBSA (From AMBER) functionalities, a well-known and excellent tool for performing end-point binding free energy calculations and to predict the stability of the complex. In this study, MM-PBSA method was employed to calculate the interaction free energy between the proteins and ligands complex under investigation.

## Results

**Fig. 2:**
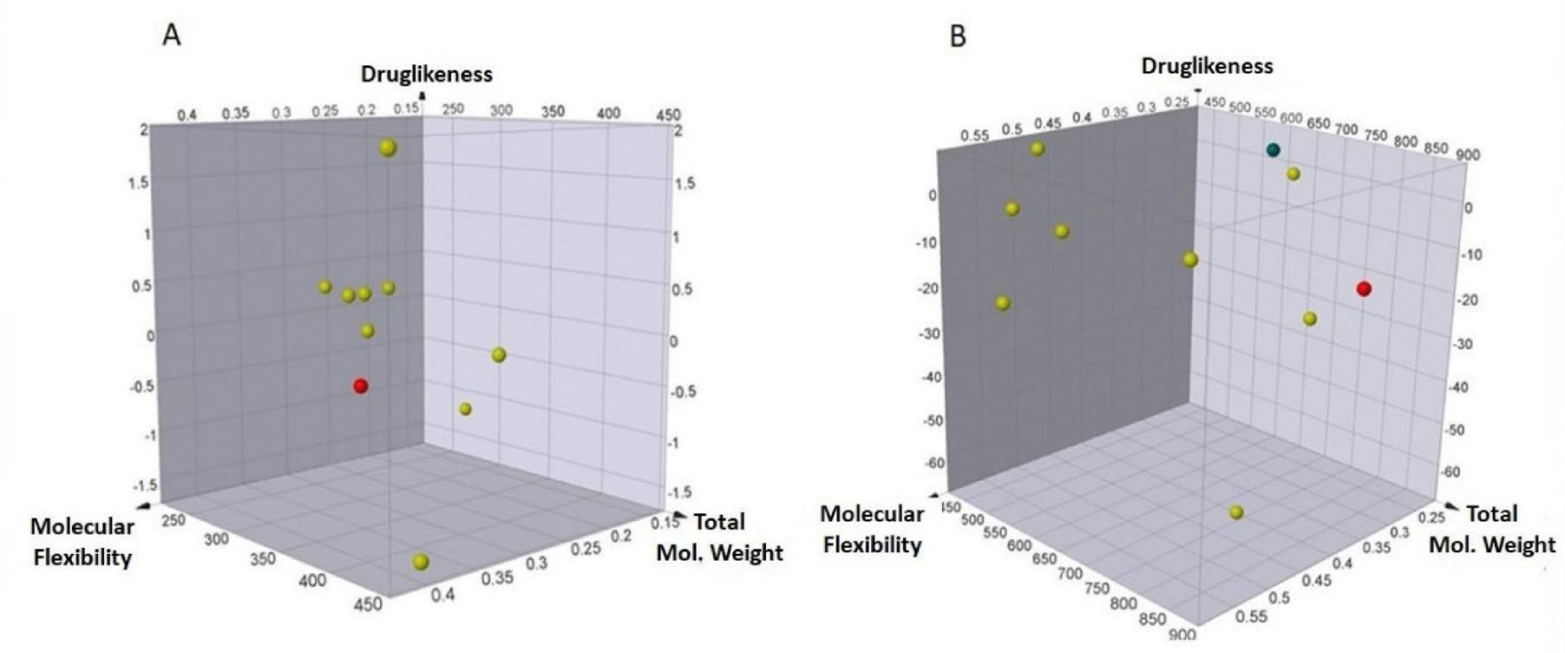
3D plot showing the druglikeness, total molecular weight, and molecular flexibility of the top ten (**A**) Phytocompounds (**B**) Drugs. The x-axis represents the druglikeness score. The y-axis shows the total molecular weight of the drugs in Daltons (Da), and the z-axis shows the molecular flexibility score. The plot was generated using Data Warrior software.

The screening process was meticulously conducted following an extensive literature search to identify 93 phytocompounds and 15 drugs with documented potential for antiviral activity. These compounds were subjected to rigorous screening using DataWarrior based on predefined criteria encompassing druglikeness, total molecular weight, and molecular flexibility to gauge their suitability as antiviral agents. The top ten drugs and phytocompounds were identified based on their optimal compliance with the screening filters (Fig. 2). These compounds emerged as promising candidates warranting further investigation in subsequent studies. The outcomes gleaned from the screening process offered valuable insights into their potential for antiviral activity, underscoring their suitability for advanced development as potential antiviral agents.

**Fig. 3:**
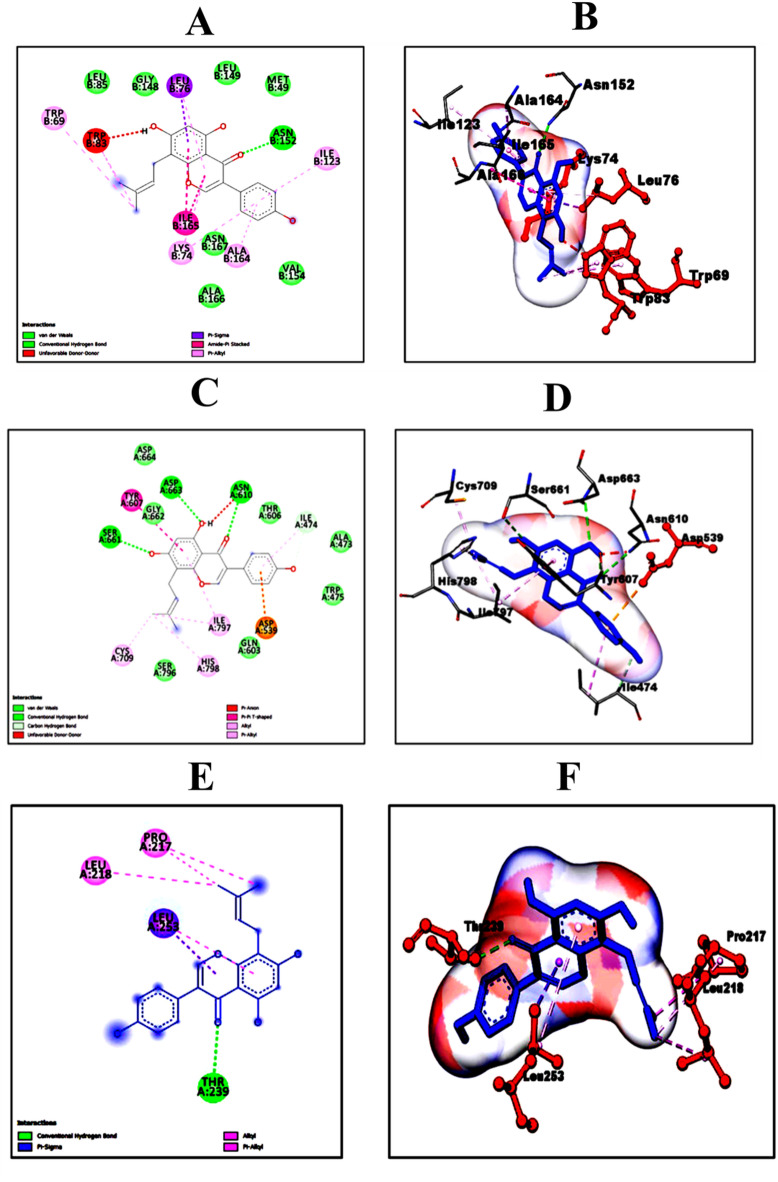
Interactions of Lupiwighteone with NS3 (**A**, **B**), NS5 (**C**, **D**) and Envelop (**E**, **F**) target proteins: (**A**, **C**, **E**) 2D interactions and (**B**, **D**, **F**) 3 D interactions.

Subsequently, docking simulations were performed utilizing AutoDockVina to assess the interactions of the top 10 phytocompounds and drugs with NS3, NS5, and E proteins of DENV. The selected phytocompounds included Aloe-emodin, Avicularin, Baicalein, Catechin, Epicatechin, Flavone, Kaempferol, Kushenol W, lupiwighteone, and Quercitrin. Remarkably, Lupiwighteone emerged as the most promising phytocompound, exhibiting average binding affinity (-8.46 kcal/mol) across all target proteins, particularly with NS3 and NS5, suggesting its potential efficacy against dengue virus infection (Table 1). The NS3 protein exhibited notable binding affinities with lupiwighteone, registering values of -8.5 kcal/mol and − 9.5 kcal/mol for DENV-2 and DENV- 4 serotypes, respectively. Following NS3, the NS5 protein of DENV- 2 and DENV- 3, as well as the E-protein of DENV- 2, also displayed considerable binding interactions with lupiwighteone. Among the 10 phytocompounds examined, lupiwighteone consistently demonstrated the highest binding affinity with all target proteins. Notably, in the case of NS3, lupiwighteone established crucial hydrogen bonding with ASN152, alongside hydrophobic interactions involving residues LEU85, GLY148, LEU76, LEU149, MET49, TRP69, TRP83, ILE123, ILE165, LYS74, ASN167, ALA164, VAL154, and ALA166 as shown Fig. 3A and B. Similarly, in interactions with NS5, lupiwighteone was observed to form hydrogen bonds with SER661, ASN610, and ASP663, while engaging in hydrophobic interactions with SER796, GLN603, ASP539, ILE474, TRP475, ALA473, THR606, TYR607, GLY662, ASP664, ILE797, HIS798, and CYS709 (refer to Fig. 3C and D). Furthermore, in its interaction with the Envelope (E) protein, lupiwighteone established hydrogen bonds with THR239 and exhibited hydrophobic interactions with LEU253, LEU218, and PRO217 (refer to Fig. 3E and F). The comprehensive summary of lupiwighteone’s interactions with the target proteins is presented in Table 3. Following lupiwighteone, Baicalein exhibited notable binding affinities with NS5 of DENV-2 and DENV-3, with binding energies of -8.2 kcal/mol and − 9.8 kcal/mol, respectively. Additionally, Baicalein demonstrated binding affinities with NS3 of DENV-2 and DENV-3, as well as with the E-protein of DENV-2, with binding energies of -8.3 kcal/mol, -7.6 kcal/mol, and − 8.7 kcal/mol, respectively. This trend was consistent with other phytocompounds, which also displayed binding affinities with all protein sequences considered in the study. However, the variations in binding scores across different serotypes raised concerns regarding their efficacy against all serotypes. Comparative analyses of docking scores were performed to contextualize the findings. Notably, lupiwighteone demonstrated higher binding affinities compared to other compounds, corroborating its potential as a lead candidate for further investigation. Moreover, the docking results were benchmarked against established standards or previous studies to validate the efficacy of the selected compounds^38^.

**Table 1:** Docking scores of 10 best phytocompounds for NS3, NS5 and E proteins.

Name of the phytoconstituents	Binding energy (kcal/mol)				
	NS3		NS5		E
	DENV-2	DENV-4	DENV-2	DENV-3	DENV-2
Lupiwighteone	-8.5	-9.5	− 8	-9.2	-7.1
Baicalein	-8.3	-7.6	-8.2	-9.8	-8.7
Epicatechin	-8.1	-8.5	-8	-8.5	-6.7
Kushenol W	-8.1	-8.9	-8.2	-8	-7.1
Aloe emodin	-8	-8.1	-7.6	-9.7	-6.6
Flavone	-8	-8.2	-8.1	-9.4	-8.2
Kaempferol	-7.7	-7.5	-7.9	-10	-6.8
Catechin	-7.6	-8.4	-8	-8.9	-6.8
Avicularin	-7.6	-8.7	-8.2	-9.3	-7.5
Quercitrin	-7.9	-9.1	-8.2	-8.6	-8.1

**Table 2:** Docking scores of 10 best drugs for NS3, NS5 and E-proteins.

Name of drug	Binding energy (kcal/mol)				
	NS3		NS5		E
	DENV-2	DENV-4	DENV-2	DENV-3	DENV-2
Bromocriptine	-11.7	-10.5	-9.2	-9.5	-7.6
Ledipasvir	-10.6	-10.6	-11.2	-9.6	-9.6
Daclatasvir	-10.2	-10.2	-8.8	-8.8	-7.6
Doravirine	-8.5	-8.7	-8.5	-9.5	-7.4
Raltegravir	-8.5	-10.2	-9.7	-9.5	-7.4
Sofosbuvir	-8.4	-8.5	-7.7	-8.6	-7.1
Grazoprevir	-8.3	-9.4	-9.7	-9.3	-7.9
Asunaprevir	-8.3	− 7.9	-8.5	-7.1	-6.4
Boceprevir	-7.9	-7.9	-7.9	-8.2	-6.7
CoPP	-7.7	-8.2	-8.5	-10.5	-7.7

**Table 3:** Interactions of Lupiwighteone with NS3, NS5 and E proteins of DENV Serotypes.

Protein	Serotype	Binding energy (kcal/mol)	Hydrogen bonds	Hydrophobic interactions
NS3	DENV-2	-8.5	ASN152	LEU85, GLY148, LEU76, LEU149, MET49, TRP69, TRP83, ILE123, ILE165, LYS74, ASN167, ALA164, VAL154, ALA166
	DENV-4	-9.5	LEU429	LYS430, GLU412, SER453, GLN456, HIS287, THR317, PRO319, ALA452, PRO318, GLY320, LYS515, SER321, THR322, TRP488, PRO451, ASP482, THR289, THR450, CYS428, ASP409
NS5	DENV-2	-8	SER661, ASN610, ASP663	SER796, GLN603, ASP539, ILE474, TRP475, ALA473, THR606, HOH1435, TYR607, GLY662, ASP664, ILE797, HIS798, CYS709
	DENV-3	-9.2	ARG361	THR362, PRO363, ARG540, GLY258, THR360, LEU257, ASP256, GLU356, LYS300, TYR119, ALA259, ALA535, VAL687
E	DENV-2	-7.1	LYS157, GLU147	HIS158, THR155, ASN153, GLY102, ASN153

The study identified a selection of top 10 drugs, comprising Asunaprevir, Boceprevir, Bromocriptine, CoPP, Daclatasvir, Doravirine, Grazoprevir, Ledipasvir, Raltegravir, and Sofosbuvir. Comprehensive data on the binding affinities of each compound with all serotypes can be found in Table 2.

### ADME prediction of drugs and phytoconstituents

To evaluate the drug-likeness of the inhibitors, all candidate molecules underwent analysis using the ProTox 3.0 server, as detailed in Tables 4 and 5. As stated above, Protox 3.0 provided insights into the compounds’ LD50 values, toxicity classes, and potential hepatotoxic, carcinogenic, mutagenic, immunotoxic, and cytotoxic activities. Additionally, Molinspiration and SwissADME^39^, both ADMET-based drug scan tools, were utilized for further assessment, as summarized in Supplementary Tables S1–S4.

**Table 4:** Toxicity prediction of 10 best phytocompounds using ProTox 3.0 server.

S.no.	Name of phytocompound	LD50 (mg/kg)	Hepato-toxicity	Carcino-genecity	Immuno-toxicity	Muta-genecity	Cyto-toxicity	Predicted toxicity class
1	Kushenol W	2000	Inactive	Inactive	Active	Inactive	Inactive	4
2	Lupiwighteone	2500	Inactive	Inactive	Inactive	Inactive	Inactive	5
3	Flavone	2500	Inactive	Active	Inactive	Inactive	Active	5
4	Baicalein	3919	Inactive	Active	Inactive	Active	Inactive	5
5	Kaempferol	3919	Inactive	Inactive	Inactive	Inactive	Inactive	5
6	Aloe emodin	5000	Inactive	Inactive	Active	Active	Inactive	5
7	Avicularin	5000	Inactive	Inactive	Active	Inactive	Inactive	5
8	Quercitrin	5000	Inactive	Active	Active	Inactive	Inactive	5
9	Catechin	10,000	Inactive	Inactive	Inactive	Inactive	Inactive	6
10	Epicatechin	10,000	Inactive	Inactive	Inactive	Inactive	Inactive	6

**Table 5:** Toxicity prediction of 10 best drugs using ProTox 3.0 server.

S.no.	Name of drug	LD50 (mg/kg)	Hepato-toxicity	Carcino-genecity	Immuno-toxicity	Muta-genecity	Cyto-toxicity	Predicted toxicity class
1	Bromocriptine	200	Inactive	Inactive	Active	Inactive	Active	3
2	Daclatasvir	200	Inactive	Inactive	Inactive	Inactive	Inactive	3
3	Raltegravir	200	Active	Inactive	Inactive	Inactive	Inactive	3
4	Ledipasvir	2000	Inactive	Inactive	Active	Inactive	Inactive	4
5	Boceprevira	1500	Inactive	Inactive	Inactive	Inactive	Inactive	4
6	CoPP	1190	Active	Inactive	Active	Inactive	Inactive	4
7	Doravirine	1000	Active	Inactive	Active	Inactive	Inactive	4
8	Grazoprevir	1000	Active	Inactive	Active	Inactive	Active	4
9	Asunaprevir	3000	Active	Inactive	Active	Inactive	Inactive	5
10	Sofosbuvir	12,000	Active	Inactive	Inactive	Inactive	Inactive	6

Among these compounds, lupiwighteone (C_20_H_18_O_5_), chemically known as 5,7-dihydroxy-3-(4-hydroxyphenyl)-8-(3-methylbut-2-enyl) chromen-4-one, exhibited notable properties. With a molecular weight of 338.36 g/mol, a miLogP value of 4.52, and a solubility of 1.008 mg/L @ 25 °C (estimated), lupiwighteone emerged as a promising phytocompound warranting further investigation.

The toxicity data obtained from the ProTox 3.0 online server revealed that all the phytocompounds were categorized under Class 4–6. Specifically, Catechin (LD50 = 10000 mg/kg), Epicatechin (LD50 = 10000 mg/kg), Kaempferol (LD50 = 3919 mg/kg), and Lupiwighteone (LD50 = 2500 mg/kg) were among the phytocompounds with no active toxicity. Furthermore, among the drugs, Bromocriptine and Daclatasvir were classified as Class 3 drugs, while Ledipasvir was categorized as Class 4. These findings provide valuable insights into the toxicity profiles of the tested compounds, guiding further investigations into their safety and efficacy for potential therapeutic applications.

The MolInspiration online server was employed to estimate the drug likeness of both the active phytocompounds and drugs. Notably, all the phytocompounds, with the exception of Quercitrin and Avicularin, were predicted to have zero violations of druglikeness rules. Among the drugs, Doravirine exhibited zero violations, indicating its potential suitability for drug development. Additionally, Raltegravir, CoPP, Bromocriptine, and Boceprevir were found to violate only one of the rules, suggesting that they are promising candidates warranting further analysis. These results highlight the druglikeliness of the tested compounds and provide valuable guidance for future investigations into their pharmacological properties and therapeutic potential.

Based on our docking results, Baicalein, KushenolW, and Lupiwighteone exhibited higher binding affinities for all the target proteins considered in our study. However, upon conducting toxicity analysis, it was revealed that Baicalein demonstrated activity for carcinogenicity and mutagenicity, while KushenolW showed activity for immunotoxicity. Consequently, these findings disqualified Baicalein and KushenolW as potential candidates for further investigation.

Notably, Lupiwighteone displayed promising ADME characteristics, further supporting its potential for MD simulation studies. Its higher binding affinity with all three target proteins of different serotypes reaffirms its potential as an effective phytocompound for combating dengue. The consistent high binding scores observed for lupiwighteone underscore its promising candidacy for therapeutic development against dengue virus infections.

Likewise, among repurposed drug candidates, although Ledipesvir showed average highest docking scores with all the serotypes, but it was found to be immunotoxic. Another drug, Bromocriptine had an average binding score of -9.7 with all proteins along all serotypes, but it was also found to be immunotoxic as well as cytotoxic. Daclatasvir was found to bind to all the proteins across serotypes efficiently with zero toxicity thus making it the best candidate for further studies.

### MD simulation of protein ligand complexes

MD simulation was done to study the stability of protein ligand complexes. Molecular docking and toxicity data revealed that lupiwighteone has the best pharmacokinetic and toxicity profile as well as excellent binding affinity for all target proteins. The complexes of the three target proteins with lupiwighteone were subjected to 100ns Molecular Dynamics simulation in a box of water to determine what the trajectory would look like and then estimate a profile of stability for the complexes and every MD simulation was done relative to a reference for each protein. Recently reported potent inhibitors were docked with the target proteins at the sites described in literature where they are thought to bind to in order to elicit their protein inhibition and the complexes obtained were subjected to MD simulation under the same conditions as the lupiwighteone complexes of the three studied proteins. The Free Energy of Binding of the complexes were also estimated using the gMMPBSA method. The Free energy of binding of the complexes showed that lupiwighteone has a very high binding energy when complexed with E-protein and NS3 protein relative to the inhibitors (Table 6). The binding energy of lupiwighteone when bound to E-protein is almost 10x that of the inhibitor and that of lupiwighteone when bound to NS3 protein is more than double that of the inhibitor used as a reference compound. However, the free energy of binding of lupiwighteone with NS5 protein is lower compared to the reference compound which shows that lupiwighteone may not be very potent against the NS5 protein compared the E-protein and NS3 protein. Though the free energy of binding of lupiwighteone with NS5 is significant, it may still be considered as an inhibitor of NS5 and this means that if lupiwighteone is administered orally as a Dengue virus therapeutic agent, it has a very high tendency of being effective at reducing the viral load because of its high potential for inhibiting three different proteins that are critical for the virus metabolism.

**Table 6:** Energy decomposition for free energy of binding (FEB) estimated for target proteins bound to respective inhibitors and lupiwighteone. VDWAALS = van der Waals energy, EEL = electrostatic energy, EPB = polar solvation energy, ENPOLAR = non-polar solvation energy, GGAS = gas-phase energy (VDWAALS + EEL), GSOLV = solvation free energy (EPB + ENPOLAR); all values in kcal/mol.

Compounds	VDWAALS (kcal/mol)	EEL (kcal/mol)	EPB (kcal/mol)	ENPOLAR (kcal/mol)	GGAS (kcal/mol)	GSOLV (kcal/mol)	Total (kcal/mol)
E-protein bound to inhibitor vs. E-protein bound to Lupiwighteone							
(1S,5R)-3-methyl-2-thioxo-1,2,3,4,5,6-hexahydro-8H-1,5-methanopyrido[1,2-a][1,5]diazocin-8-one (E-protein inhibitor)	-2.89 ± 1.85	-1.04 ± 0.9	2.31 ± 1.7	-0.34 ± 0.22	-3.94 ± 2.63	1.96 ± 1.48	-1.98 ± 1.21
Lupiwighteone bound to E-protein	-35.66 ± 1.03	-5.48 ± 0.88	31.42 ± 1.23	-3.98 ± 0.07	-41.14 ± 1.21	27.44 ± 1.21	-13.7 ± 1.5
NS3 protein bound to inhibitor vs. NS3 bound to Lupiwighteone							
(2S,4S)-4-(2-Hydroxyphenyl)-2-(methylthio)-1,2,3,4-tetrahydropyrrolo[1,2-a]quinolin-3-one (NS3 inhibitor)	-16.04 ± 2.34	-2.62 ± 1.22	13.44 ± 2.56	-1.88 ± 0.24	-18.66 ± 2.95	11.55 ± 2.34	-7.11 ± 1
Lupiwighteone bound to NS3	-34.09 ± 3.41	-12.09 ± 1.95	34.26 ± 3.62	-3.51 ± 0.34	-46.18 ± 4.66	30.75 ± 3.31	-15.43 ± 1.68
NS5 protein bound to inhibitor vs. NS5 bound to Lupiwighteone							
2-Oxo-1,3-benzoxathiol-5-yl acetate (NS5 inhibitor)	-22.52 ± 0.51	-5.01 ± 2.47	19.75 ± 2.37	-2.55 ± 0.05	-27.53 ± 2.56	17.21 ± 2.38	-10.32 ± 1.04
Lupiwighteone bound to NS5	-34.72 ± 0.9	-14.31 ± 1.89	48.34 ± 1.39	-3.92 ± 0.04	-49.03 ± 2.56	44.42 ± 1.38	-4.61 ± 2.31

**Fig. 4:**
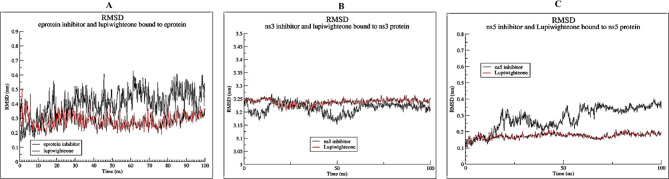
RMSD (**A**) E-protein inhibitor and Lupiwighteone bound to E-protein, (**B**) NS3 inhibitor and Lupiwighteone bound to NS3, (**C**) NS5 inhibitor and Lupiwighteone bound to NS5 protein.

**Fig. 5:**
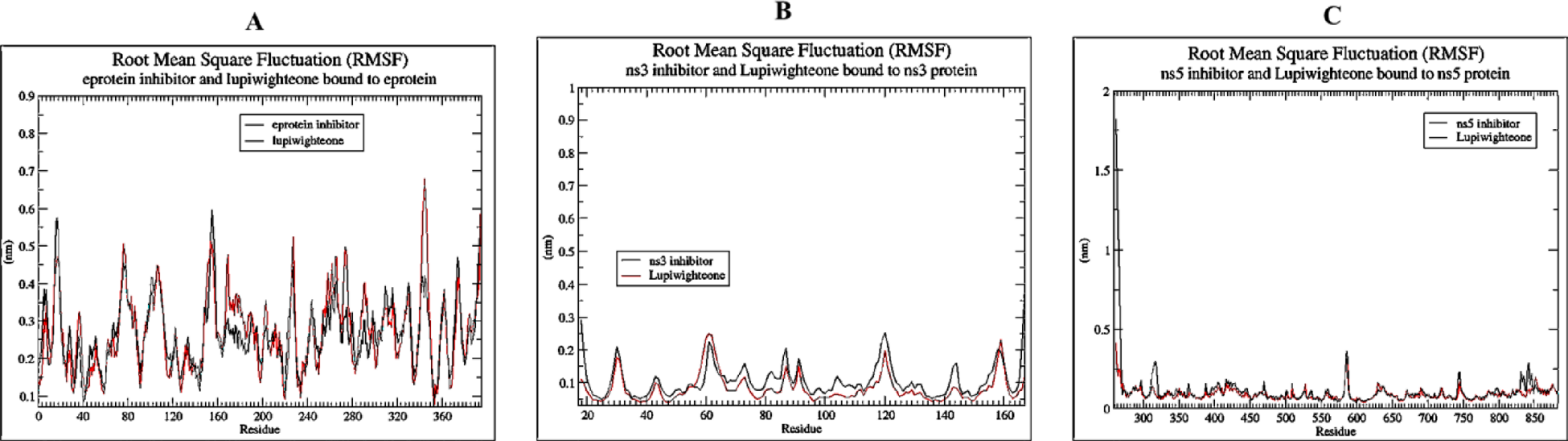
RMSF (**A**) E-protein inhibitor and Lupiwighteone bound to E-protein, (**B**) NS3 inhibitor and Lupiwighteone bound to NS3, (**C**) NS5 inhibitor and Lupiwighteone bound to NS5 proteins.

**Fig. 6:**
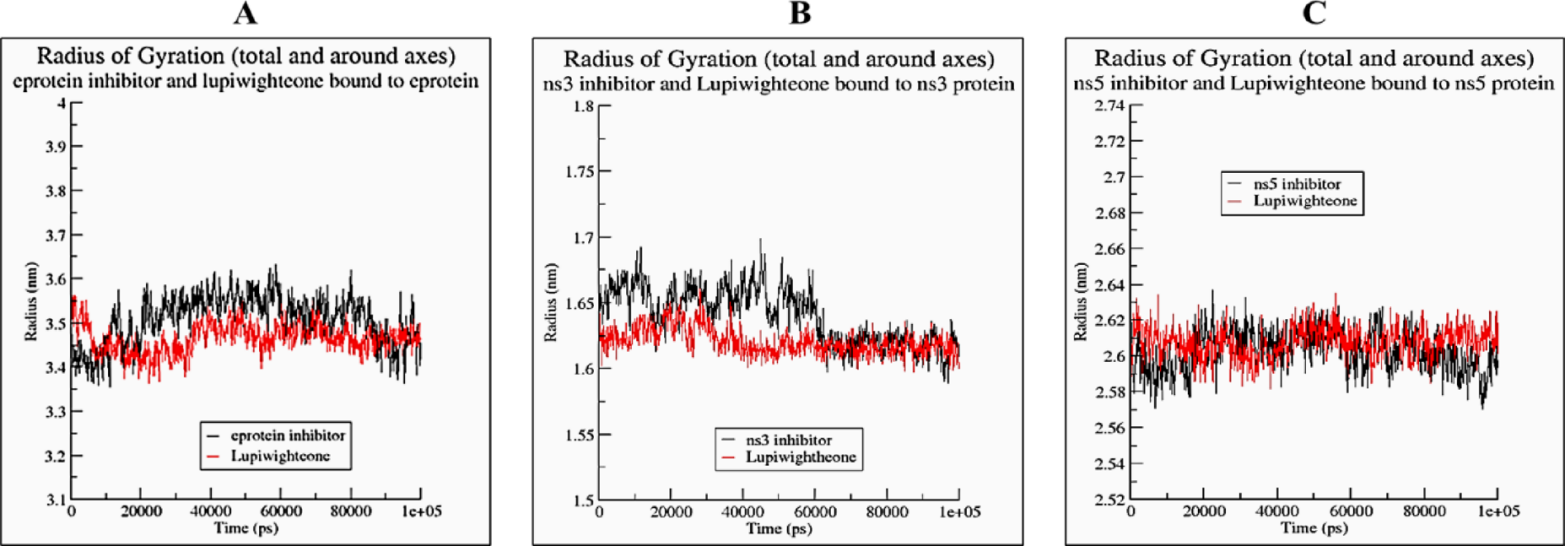
Radius of gyration (**A**) E-protein inhibitor and Lupiwighteone bound to E-protein, (**B**) NS3 inhibitor and Lupiwighteone bound to NS3, (**C**) NS5 inhibitor and Lupiwighteone bound to NS5 proteins.

**Fig. 7:**
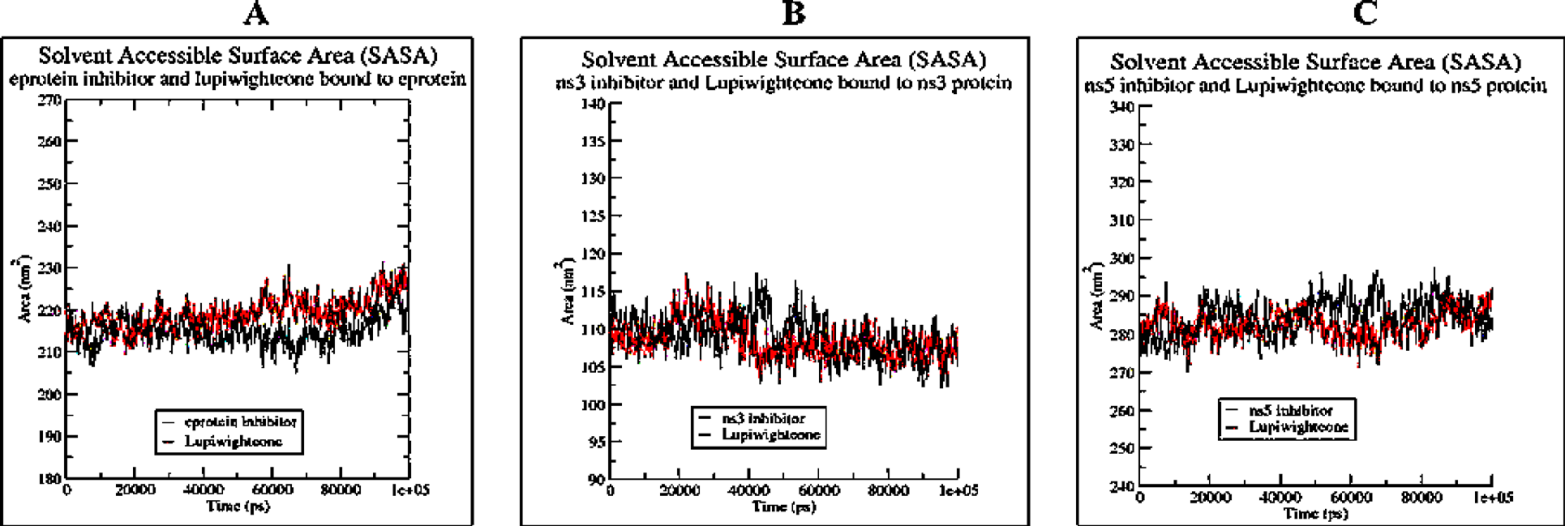
Solvent Accessible Surface Area (SASA) (**A**) E-protein inhibitor and Lupiwighteone bound to E-protein, (**B**) NS3 inhibitor and Lupiwighteone bound to NS3, (**C**) NS5 inhibitor and Lupiwighteone bound to NS5 proteins.

**Fig. 8:**
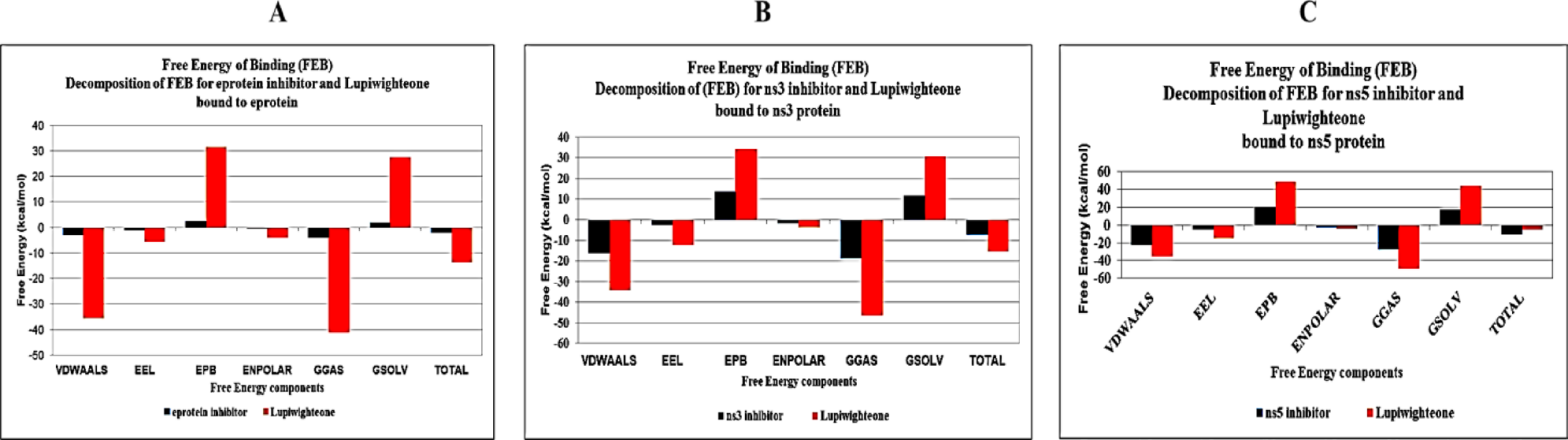
Estimated Binding free energy Breakdown (gMMPBSA): (**A**) E-protein, (**B**) NS3 and (**C**) NS5 proteins in complex with lupiwighteone and corresponding inhibitors.

The RMSD plots (Fig. 4) of the complex of lupiwighteone and the studied proteins also show that the complexes are stable within the 100ns time frame for which the complexes were studied in the molecular dynamics simulation. The complexes of lupiwighteone achieved equilibrium shortly after the commencement of the simulation relative to the complexes of the inhibitors for the three studied proteins. The RMSF plots (Fig. 5) of the three studied proteins bound with lupiwighteone and corresponding inhibitor shows that the fluctuations of amino acid residues in the site to which the ligands bound are similar in all cases for individual proteins except for E-protein where residues in the range of 330–340 fluctuated more intensely when bound to lupiwighteone compared to when it was bound to the E-protein inhibitor. The plots of radius of gyration as well as solvent accessible surface area (Figs. 6 and 7) also showed that the proteins were not overly perturbed by the lupiwighteone during the course of the simulation (very little conformational changes) which suggests that the protein maintained a stable confirmation while bound to the lupiwighteone. Essentially, the plots from the trajectory obtained from the MD simulation showed that the complexes obtained for lupiwighteone bound to the target proteins were stable and the estimated Free Energy of Binding (FEB) suggests that lupiwighteone is worth considering for a follow up biological study involving the target proteins directly or an animal model (Fig. 8).

## Discussion

The evolution of dengue virus and the emergence of its four distinct serotypes (DENV1-4) have been the subject of much research and interest in the scientific community. Despite the earliest recorded dengue epidemics occurring in the 18th and 19th centuries^40^, the virus is thought to have originated even earlier, with evidence suggesting it may have circulated in primates for thousands of years. The diversification of the virus into multiple serotypes is attributed to genetic drift and to some extent recombination events that occurred during its evolutionary journey, as it spread to new regions and encountered different host populations^41^. The circulation of these serotypes presents a significant challenge for controlling the spread of the disease and developing effective vaccines and drugs^42^.

The main challenge in vaccine development is to create a tetravalent formulation such that it has equally effective immunization for all the four DENV serotypes^43^. Even though vaccine development for dengue started in the 1920s, till date around 7 vaccines have gone through various clinical trials^44^. The first commercialized and FDA approved vaccine, Dengvaxia^®^ (CYD-TDV) is currently available and licensed in 20 countries but it has raised some safety issues which even lead to retraction of Dengvaxia’s license in some countries including Philippines^8^. Despite these setbacks, two other vaccine candidates, LAV-TDV from NIAID/Butantan and TAK-003 from Takeda, have shown promising results in phase III clinical trials. However, concerns about vaccine efficacy and safety persist, particularly in light of historical immunogenicity data and recent disclosures of variable efficacy against all DENV serotypes. Previous study underscores the importance of prioritizing vaccine formulations that encompass a comprehensive array of epitopes essential for inducing protective immunity, spanning both cellular and humoral responses. This imperative remains relevant despite comparable population coverage percentages achieved by LAV-TDV and TAK-003 vaccine formulations^45,46^.

In light of these challenges, immunoinformatics has emerged as a pivotal tool in designing multi-epitope vaccines capable of targeting all DENV serotypes. Recent research highlights the potential of immunoinformatics to identify conserved epitopes from structural and non-structural E-protein, essential for robust immune responses. Kaushik et al. designed a candidate vaccine targeting DENV-1 to DENV-4 through conserved epitopes, demonstrating its immunogenicity and antibody production in vivo. These findings underscore the importance of immunoinformatics in developing next-generation vaccines capable of providing comprehensive protection against dengue virus^47^.

The drug discovery for dengue had commenced decades ago but is not an easy process. The lack of a potent drug covering all serotypes signifies that more resources need to be explored. Firstly, an anti-DENV drug should be inhibitory to all the serotypes and even combat the antibody dependent enhancement which is seen during secondary dengue infection^48^. Secondly, drug development in general is a long and tedious process making it tough to develop a de novo drug. Hence, repurposing of drugs comes into picture. This strategy reduces the time and expense of drug development because the majority of the pharmaceuticals that are being repurposed are already FDA-authorized thereby expediting the process^49^.Therefore, we conducted a comparative study to find a common repurposed drug which will be effective against the three most common and threatening serotypes of dengue including DENV-2, DENV-3 and DENV-4^50^.

Based on comparative analysis of the binding affinities, our data indicates that three drugs including Daclatasvir, Ledipasvir and Bromocriptine can serve as potential candidates for inhibiting the selected target proteins of Dengue virus (NS3, NS5 and E-protein). Interestingly, Daclatasvir and Ledipasvir were reported to belong to the class of FDA approved direct-acting antiviral drugs (DAA) which were previously used in the treatment of Hepatitis C (phylogenetically related to DENV)^51,52^. The role of Bromocriptine against DENV has also been shown in one of the previous studies^53^, and our comparative analysis justifies its use against other DENV serotypes as well.

A previous study also reported Daclatasvir to be an effective agent against the E-protein of DENV-2^54^. Our analysis supports their finding and also indicates the effectiveness of this drug against NS3 of DENV-2 and DENV-4 (binding affinity of -10.2 kcal/mol each) and NS5 protein of DENV-2 and DENV-3 (binding affinities of -8.8 kcal/mol each). Ledipasvir was found to have consistently high binding affinities with all three conserved proteins (NS5, NS3 and E proteins). Ledipasvir is FDA-approved direct-acting antiviral drug (DAA) recommended for viral infections like HCV^53,55^ and COVID-19^56–58^. It has shown little to no side effects on patients. Our analysis indicates the effectiveness of this drug against NS3 of DENV-2 and DENV-4 (binding affinity of -10.6 kcal/mol each) and NS5 protein of DENV-2 and DENV-3 (binding affinities of -11.2 kcal/mol and −9.6 kcal/mol respectively). Apart from the two drugs discussed above, Bromocriptine also showed consistent high binding affinities with NS3 protein of DENV-2 and DENV- 4 (binding affinities of -11.7 and −10.2 kcal/mol) and NS5 for DENV-2 and DENV-3. This is also supported by an in vitro study where bromocriptine has been shown to have potent anti-dengue virus (DENV) activity and low cytotoxicity^53^. Thus, we suggest in vitro as well as in vivo analysis of Daclatasvir, Ledipasvir and Bromocriptine as drug candidates for treating Dengue infection and preventing Antibody (Ab)-dependent enhancement of infection.

While the enlisted synthetic compounds showed encouraging results against the targeted proteins from the *insilico* study, because they are known drugs being used clinically already, there is plenty of documentation about their side effects and actual toxicity profile. Declatasvir, though shows successful all-oral regimens of antiviral therapy in patients with chronic hepatitis C and cirrhosis is occasionally complicated by hepatic decompensation (chronic liver injury) and may cause reactivation of hepatitis B in susceptible patients coinfected with the hepatitis B virus (HBV). Ledipasvir has also been linked to an adverse effect that causes lung diseases^59^ just as bromocriptine has been found to have potential for liver injury (especially when overdosed which happens accidentally in children). Our pharmacokinetics and toxicity profile predictions also show that the synthetic compounds may be toxic when administered especially at high doses.

On the other hand, the phytochemical, lupiwighteone (8-prenylgenistein), a naturally occurring polyphenol-based phytocompound caught our attention. This pyrano-isoflavone compound derived from *Erythrina variegate* (the Indian Coral TrADEee)^60^ has exhibited remarkable binding affinities with all the target proteins, indicating its potential role as an inhibitor. Lupiwighteone demonstrates extraordinary organic properties which makes it the finest prospective compound for therapeutical purposes in our list. It belongs to toxicity class 5 and was observed to be negative for all toxicity profiles on ProTox 3.0 analysis with LD50 as high as 2500 mg/kg. Lupiwighteone has high affinity with NS5 of DENV-2 and DENV-3; NS3 of DENV-2 and DENV-4 and E-protein of DENV-2. Additionally, it follows Lipinski’s rule of five with no violations, indicating the highest druglikeliness. The MD Simulation results underscore the potential of lupiwighteone as a lead candidate for antiviral drug development against dengue virus.

The findings of this study not only unravel the molecular mechanisms governing the interaction between lupiwighteone and target proteins but also offer valuable insights into its pharmacological profile. These results set the stage for subsequent experimental validation and clinical translation, addressing the critical need for effective antiviral therapies against dengue virus infections. Therefore, based on this in silico comparative analysis, we propose lupiwighteone as a prospective phytocompound for the treatment of dengue infection, potentially exhibiting inhibitory effects against DENV-2, DENV-3, and DENV-4 serotypes. Leveraging computational methodologies and advanced molecular modeling techniques, this research contributes to ongoing endeavors in drug discovery and development to combat dengue virus infections. By conducting a comparative analysis of promising phytocompounds and drugs against different serotypes, the present study offers a rational approach to identifying broad-spectrum therapeutics.

## Conclusion

Amidst the alarming statistics provided by the World Health Organization (WHO), which indicate that roughly half of the global population is now vulnerable to Dengue virus (DENV) infection, with an estimated 100–400 million cases reported annually, the urgency to combat this pervasive threat has reached unprecedented levels. Despite the significant global burden of Dengue, there remains a lack of anti-DENV antivirals, prophylactics, or therapeutics availability. Addressing this pressing global health challenge, our study pioneers a coherent approach to identify potential therapeutics against DENV. Through meticulous screening, integrating molecular docking studies, ADMET analysis, and druglikeness profiling, we have uncovered promising repurposed drugs and phytocompounds exhibiting consistent binding affinity across DENV serotypes. In summary, our study underscores the promising antiviral activity of phytocompound lupiwighteone and also highlights the candidature of daclatasvir as repurposed drug which calls for further exploration of its therapeutic potential. These novel findings not only offer a ray of hope in the battle against DENV but also set the stage for further experimental validation and clinical translation, promising a brighter future in the fight against Dengue infections.

## Electronic supplementary material

Below is the link to the electronic supplementary material.

Supplementary Material 1

## Data Availability

All data generated or analysed during this study are included in this published article. Additional data will be made available on a reasonable request.

## Abbreviations

ADMET: Absorption, distribution, metabolism, excretion, and toxicity
LD50: Lethal dose 50
MD Simulation: Molecular dynamics simulation
MMPBSA: Molecular mechanics Poisson Boltzmann surface area
NCBI: National Center of Biotechnology Information
PDB: Protein data bank
RMSD: Root mean square deviation
RMSF: Root mean square fluctuations
ROG: Radius of gyration
SASA: Solvent accessible surface area

## Declarations

### Human and animals rights

This research article does not contain any studies with human participants or animals performed by any of the authors.
