# Correction: Comparative analysis of phytocompounds and repurposed drugs against dengue virus serotypes employing an in silico study

**DOI:** 10.1038/s41598-025-32110-x

**Published:** 2025-12-23

**Authors:** Pranjal Vats, Rajan Rolta, Deeksha Salaria, Ishika Sharma, Anita Verma, Olatomide A. Fadare, Mansi Verma

**Affiliations:** 1 School of Biological Sciences, The University of Manchester, Oxford Road, Manchester, M13 9PL UK; 2 Department of Pharmacology, Post Graduate Institute of Medical Education and Research, Chandigarh, 160012 India; 3 Sri Venkateswara College, University of Delhi, New Delhi, 110021 India; 4 Organic Chemistry Research Lab, Department of Chemistry, ObafemiAwolowo University, Ile-Ife, Osun 220282 Nigeria; 5 Department of Zoology, Hansraj College, University of Delhi, Delhi, 110007 India

Correction to: *Scientific Reports* 10.1038/s41598-025-06974-y, published online 30 July 2025

In the original version of this Article, the abbreviation “MMPBSA” was incorrectly given as “MMBPSA”.

In addition, in the Methods section, the subheading ‘Virtual screening and molecular Docking’ has been corrected to “Molecular Docking”.

Under the subheading ‘In silico toxicity prediction of phytocompounds and drugs’,

“The analysis utilized several tools, including SwissADME (http://www.swissadme.ch), MolInspirationCheminformatics (https://www.molinspiration.com), and PROTOX 3.0 (https://tox-new.charite.de/protox_II/).”

now reads:

“The analysis utilized several tools, including SwissADME (http://www.swissadme.ch), MolInspirationCheminformatics (https://www.molinspiration.com), and ProTox 3.0 (https://tox.charite.de/protox3/).”

Under the subheading ‘Molecular dynamics (MD) simulation’,

 “For long-range electrostatics, Particle-mesh Ewald (PME) algorithm was used. The snapshots of poses from the last frame of the trajectory are presented in Fig. 3. After successful completion of molecular dynamic simulation for 100 ns, Root mean square deviation (RMSD) of backbone residues, number of Hydrogen bonds, Root mean square fluctuations (RMSF), Radius of gyration (RG) and solvent accessible surface area (SASA)were calculated from the trajectory files from each complex.”

now reads:

“For long-range electrostatics, Particle-mesh Ewald (PME) algorithm was used. After successful completion of molecular dynamic simulation for 100 ns, Root mean square deviation (RMSD) of backbone residues, number of Hydrogen bonds, Root mean square fluctuations (RMSF), Radius of gyration (RG) and solvent accessible surface area (SASA)were calculated from the trajectory files from each complex.”

In the Results section,

“Comprehensive data on the binding affinities of each compound with all serotypes can be found in Table 2 for detailed reference (Table 3).”

now reads:

“Comprehensive data on the binding affinities of each compound with all serotypes can be found in Table 2.”

Furthermore, the caption of Table 6 was incomplete.

“Energy decomposition for free energy of binding (FEB) estimated for target proteins bound to respective inhibitors and lupiwighteone.”

now reads:

“Energy decomposition for free energy of binding (FEB) estimated for target proteins bound to respective inhibitors and lupiwighteone. VDWAALS = van der Waals energy, EEL = electrostatic energy, EPB = polar solvation energy, ENPOLAR = non-polar solvation energy, GGAS = gas-phase energy (VDWAALS + EEL), GSOLV = solvation free energy (EPB + ENPOLAR); all values in kcal/mol.”

The original Article has been corrected.

