# Supplementary material for: Comparative analysis of phytocompounds and repurposed drugs against dengue virus serotypes employing an in silico study
Source: Sci Rep. 2025 Jul 30;15:27878. doi: 10.1038/s41598-025-06974-y (PMC12311024; doi:10.1038/s41598-025-06974-y)
Supplement: Supplementary file 1 — Supplementary Material 1 [file 41598_2025_6974_MOESM1_ESM.docx]

**Supplement Tables**

**Table S1: ADME prediction of 10 best phytocompounds by SwissADME server**

| **S.NO.** | **Name of Phytocompound** | **Consensus Log Po/w** | **Water Solubility** | **GI Absorption** | **TPSA** | **Lipinski Rule** | **Ghose Rule** | **Veber Rule** | **Egan Rule** | **Muegge Rule** | **Bioavailability Score** |
| --- | --- | --- | --- | --- | --- | --- | --- | --- | --- | --- | --- |
| **1** | Aloe emodin | 1.5 | Soluble | High | 94.83 | Yes; 0 violation | Yes | Yes | Yes | Yes | 0.55 |
| **2** | Avicularin | 0.19 | Soluble | low | 190.28 Å² | No; 2 violations: NorO>10, NHorOH>5 | yes | No; 1 violation: TPSA>140 | No; 1 violation: TPSA>131.6 | No; 3 violations: TPSA>150, H-acc>10, H-don>5 | 0.17 |
| **3** | Baicalein | 2.24 | Moderately soluble | High | 90.9 | Yes; 0 violation | Yes | Yes | Yes | Yes | 0.55 |
| **4** | Catechin | 0.83 | Soluble | High | 110.38 Å² | Yes; 0 violation | Yes | Yes | Yes | Yes | 0.55 |
| **5** | Epicatechin | 0.85 | Soluble | High | 110.38 | Yes; 0 violation | Yes | Yes | Yes | Yes | 0.55 |
| **6** | FLAVONE | 3.18 | Poorly soluble | High | 30.21 Å² | Yes; 0 violation | Yes | Yes | Yes | Yes | 0.55 |
| **7** | Kaempferol | 1.58 | Soluble | High | 111.13 Å² | Yes; 0 violation | Yes | Yes | Yes | Yes | 0.55 |
| **8** | Kushenol W | 2.85 | Moderately soluble | High | 116.45 | Yes; 0 violation | Yes | Yes | Yes | Yes | 0.55 |
| **9** | Lupiwighteone | 3.51 | Moderately soluble | High | 90.9 | Yes; 0 violation | Yes | Yes | Yes | Yes | 0.55 |
| **10** | Quercitrin | 0.16 | Soluble | low | 190.28 Å² | No; 2 violations: NorO>10, NHorOH>5 | yes | No; 1 violation: TPSA>140 | No; 1 violation: TPSA>131.6 | No; 3 violations: TPSA>150, H-acc>10, H-don>5 | 0.17 |

**Table S2: ADME prediction of 10 best drugs by SwissADME server**

| **S.No.** | **Name of Drugs** | **Consensus Log Po/w** | **Water Solubility** | **GI Absorption** | **TPSA** | **Lipinski Rule** | **Ghose Rule** | **Veber Rule** | **Egan Rule** | **Muegge Rule** | **Bioavailability Score** |
| --- | --- | --- | --- | --- | --- | --- | --- | --- | --- | --- | --- |
| **1** | Asunaprevir | 3.67 | poorly soluble | low | 190.71 Å² | No; 2 violations: MW>500, NorO>10 | No; 3 violations: MW>480, MR>130, #atoms>70 | No; 2 violations: Rotors>10, TPSA>140 | No; 1 violation: TPSA>131.6 | No; 3 violations: MW>600, TPSA>150, Rotors>15 | 0.17 |
| **2** | boceprevira | 2.09 | Soluble | Low | 150.7 | Yes; 1 violation | No; 3 violations | No; 2 violations | No; 1 violation | No; 1 violation | 0.55 |
| **3** | Bromocriptine | 3.11 | Poorly soluble | High | 118.21 | Yes; 1 violation | No; 3 violations | Yes | Yes | No; 1 violation | 0.55 |
| **4** | CoPP | 2.1 | Soluble | Low | 150.70 Å² | Yes; 1 violation: MW>500 | No; 3 violations: MW>480, MR>130, #atoms>70 | No; 2 violations: Rotors>10, TPSA>140 | No; 1 violation: TPSA>131.6 | No; 1 violation: TPSA>150 | 0.55 |
| **5** | daclatasvir | 4.06 | Poorly soluble | Low | 174.64 | No; 2 violations | No; 3 violations | No; 2 violations | No; 1 violation: | No; 4 violations | 0.17 |
| **6** | Doravirine | 2.75 | Soluble | High | 105.70 Å² | Yes; 0 violation | Yes | Yes | Yes | Yes | 0.55 |
| **7** | grazoprevir | 3.19 | Poorly soluble | Low | 203.6 | No; 2 violations | No; 3 violations | No; 1 violation | No; 1 violation | No; 3 violation | 0.17 |
| **8** | Ledipasvir | 6.44 | poorly soluble | low | 174.64 Å² | No; 2 violations: MW>500, NorO>10 | No; 4 violations: MW>480, WLOGP>5.6, MR>130, #atoms>70 | No; 2 violations: Rotors>10, TPSA>140 | No; 2 violations: WLOGP>5.88, TPSA>131.6 | No; 5 violations: MW>600, XLOGP3>5, TPSA>150, #rings>7, Rotors>15 | 0.17 |
| **9** | Raltegravir | 1.46 | Soluble | low | 152.24 Å² | Yes; 1 violation: NorO>10 | Yes | No; 1 violation: TPSA>140 | No; 1 violation: TPSA>131.6 | No; 1 violation: TPSA>150 | 0.55 |
| **10** | sofosbuvir | 1.48 | Moderately soluble | Low | 167.99 | No; 2 violations: | No; 1 violation | No; 2 violations | No; 1 violation | No; 2 violations | 0.17 |

**Table S3: Drug likeliness prediction of 10 best phytocompounds using MolInspiration**

| **S.No.** | **Name of Phytocompound** | [**miLogP**](https://www.molinspiration.com/services/logp.html) | [**TPSA**](https://www.molinspiration.com/services/psa.html) | **natoms** | **MW** | **nON** | **nOHNH** | **Nviolations** |
| --- | --- | --- | --- | --- | --- | --- | --- | --- |
| **1** | Aloe emodin | 2.42 | 94.83 | 20 | 270.24 | 5 | 3 | 0 |
| **2** | Avicularin | 0.8 | 190.28 | 31 | 434.35 | 11 | 7 | 2 |
| **3** | Baicalein | 2.68 | 90.89 | 20 | 270.24 | 5 | 3 | 0 |
| **4** | Catechin | 1.37 | 110.37 | 21 | 290.27 | 6 | 5 | 0 |
| **5** | Epicatechin | 1.37 | 110.37 | 21 | 290.27 | 6 | 5 | 0 |
| **6** | Flavone | 3.74 | 30.21 | 17 | 222.24 | 2 | 0 | 0 |
| **7** | Kaempferol | 2.17 | 111.12 | 21 | 286.24 | 6 | 4 | 0 |
| **8** | Kushenol W | 4.1 | 116.45 | 28 | 386.4 | 7 | 4 | 0 |
| **9** | Lupiwighteone | 4.52 | 90.89 | 25 | 338.36 | 5 | 3 | 0 |
| **10** | Quercitrin | 0.64 | 190.28 | 32 | 448.38 | 11 | 7 | 2 |

**Table S4: Drug likeliness prediction of 10 best drugs using MolInspiration**

| **S.No.** | **Name of Drug** | [**miLogP**](https://www.molinspiration.com/services/logp.html) | [**TPSA**](https://www.molinspiration.com/services/psa.html) | **natoms** | **MW** | **nON** | **nOHNH** | **Nviolations** |
| --- | --- | --- | --- | --- | --- | --- | --- | --- |
| **1** | Asunaprevir | 6.12 | 182.34 | 51 | 748.3 | 14 | 3 | 3 |
| **2** | Boceprevira | 1.8 | 150.7 | 37 | 519.69 | 10 | 5 | 2 |
| **3** | Bromocriptine | 3.6 | 118.21 | 43 | 654.61 | 10 | 3 | 1 |
| **4** | CoPP | 4.53 | 128.58 | 42 | 560.65 | 8 | 2 | 1 |
| **5** | Daclatasvir | 7.77 | 174.65 | 54 | 738.89 | 14 | 4 | 3 |
| **6** | Doravirine | 2.08 | 105.72 | 29 | 425.75 | 8 | 1 | 0 |
| **7** | Grazoprevir | 6.22 | 195.23 | 54 | 766.92 | 15 | 3 | 3 |
| **8** | Ledipasvir | 9.21 | 174.65 | 65 | 889.02 | 14 | 4 | 3 |
| **9** | Raltegravir | 0.81 | 152.25 | 32 | 444.42 | 11 | 3 | 1 |
| **10** | Sofosbuvir | 0.64 | 158.2 | 36 | 529.46 | 12 | 3 | 2 |
